# Analytical parameters and vital signs in patients subjected to dental extraction

**DOI:** 10.4317/jced.53474

**Published:** 2017-02-01

**Authors:** Enric Jané-Pallí, Carlos Arranz-Obispo, Beatriz González-Navarro, Jesús Murat, Raúl Ayuso-Montero, Santiago Rojas, Amparo Santamaría, Enric Jané-Salas, José López-López

**Affiliations:** 1 Departament of Odontostomatology. School of Dentistry. Barcelona University. Hospitalet de Llobregat, Spain; 2 Thrombotargets Europe, S.L. Mediterranean Technological Park (Parque Mediterráneo de la Tecnología). Castelldefels, Spain; 3 Hemostasis and Thrombosis Unit. Department of Hematology. Hospital Universitario Vall d’Hebrón. Barcelona, Spain

## Abstract

**Background:**

Dental consultation may provoke stress to the patient, especially when a dental surgery is going to be performed, stressful situations can cause a reaction in the sympathetic nervous system that could lead to cardiovascular alterations. Blood pressure and cardiac frequency are used often as an indirect measurement and this parameters combined can serve as good indicators of stress. Objective: Analyze the changes in vital signs and analytical parameters induced by a dental extraction.

**Material and Methods:**

24 healthy patients who required a simple dental extraction underwent to a blood test and motorization of their pre- and post-extraction vital signs before, at 2 and 48 hours after the procedure. Data analysis was performed by means of repeated measures one way ANOVA followed by multiple comparisons Bonferroni’s Post-hoc test.

**Results:**

The evaluated patients were 13 women and 11 men with an average age of 35.1. Thirteen patients (54.17% of the sample) were smokers and five were regular drinkers (20.8%). No significant differences were observed in the vital signs with the exception of diastolic blood pressure and cardiac rate that were slightly lower after extraction. Only two analytical parameters showed statistical significant changes. Total bilirubin was significantly higher at 48 hours after extraction and leukocyte count was significantly lower at this time. In any case, the magnitude of the changes observed was very low. The analytical parameters and the vital signs did not show any relevant change.

**Conclusions:**

Eventual alterations found after simple tooth extraction should not be attributed to the procedure.

** Key words:**Blood pressure, heart rate, monitoring physiologic, oxygen saturation, tooth extraction.

## Introduction

One of the objectives of dentistry is to preserve the teeth, but in determined situations dental extraction is necessary (1,2). Thus, in spite of the significant advances in odontology, dental extraction continues to be the most common procedure in dental consultations and two of the causes for such extraction, periodontal disease and caries, are among the four diseases with the highest economic cost (2,3). On the other hand, we must not forget that dental extraction is a surgical procedure that provokes fear and phobia in patients. However if the technique is adequate, the complications are minimal. It is always necessary to obtain a proper medical history that helps to minimize complications such as: postoperative pain, bleeding, inflammation, as well as difficulty in healing, which have been constantly referenced throughout literature. In this way, it was reported that up to 34.2% of patients have experienced changes in their quality of life after a simple dental extraction (4).

Excessive bleeding after a dental extraction is a relevant complication that can occur even after a routine tooth extraction, especially if the patient presents hemostatic alterations (5,6). However, at times such bleeding is unexpected, or very occasionally it is associated with the use of anti-inflammatory drugs (7,8).

Aside of bleeding and pain, another frequent complication of dental treatments are the occurrence of cardiovascular changes, which are usually harmless in healthy subjects, but can be harmful in those with previous pathology, especially in the case of heart disease (9). The mere dental consultation may provoke stress to the patient and therefore cause a reaction in the sympathetic nervous system that could lead to cardiovascular alterations (10). If a risk for the patient is anticipated, the professional must stop the treatment. However, in order to do so, it is necessary to have well established alert parameters. Blood pressure and cardiac frequency are often used as an indirect measurement of that problem, but their usefulness for this purpose was not yet completely established (11,12). Most studies indicate that both parameters combined can serve as good indicators of stress provoked by a dental consultation especially if the dentist must perform even the simplest surgical procedure (13). With respect to routine interventions, such as dental extraction, it is rare to monitor the vital signs of healthy patients or to perform blood tests. However, some studies have linked the changes in vital signs with possible stress and the use of determined anesthetics (14,15). To our knowledge, no other reported studies have performed pre- and post-extraction blood test or have monitored the oxygen saturation levels. It can be hypothesized that although it is a minor surgical procedure, dental extraction could induce changes in some analytical parameters such as D-dimer or leucocyte count.

Our objective in the present study was to examine the changes in the vital signs and analytical parameters induced by a simple dental extraction.

## Material and Methods

The data of this research were obtained during the realization of the TETIS study (NCT01595360), whose main objective was to evaluate the safety and tolerability of the product TT-173 when it was applied to the bleeding alveolus after dental extraction (Thrombotargets Europe, S.L. Spain. Protocol: THR-TT173-2010-02/ EudraCT: 2010-021882-57). Since treatment did not induce significant changes in vital signs or analytical parameters, the data of both groups were pooled and analyzed in order to determine the changes induced by tooth extraction.

-Patients and parameters evaluated

Twenty four adult volunteers of both sexes were recruited in this study. Vital signs and analytical determinations were obtained before extraction and after 2 and 48 hours. The parameters evaluated include cardiac frequency, systolic and diastolic blood pressure, temperature, oxygen saturation, complete hemogram, aPTT, PT, thrombin time, fibrinogen concentration, D-dimer, AST, ALT, total bilirubin, Ca, Cl, alkaline phosphatase, GGT, LDH, K, Na, urea, creatinine, urate, total proteins and albumin.

-Protocol for the Dental Extraction

The simple dental extraction was performed with anesthesia without vasoconstrictor (Mepivacaine 3%) applied periapically in the upper jaw or blocking the nerve in the mandible. After the extraction the integrity of the tooth was assessed and the alveolus was checked by curettage, of its four walls, as well as the apex area.

-Statistical Analysis

Data analysis was performed by means of repeated measures One way ANOVA followed by multiple comparisons Bonferroni’s Post-hoc test. The significance level for all of the statistical tests was established at a value of *p* < 0.05.

-Ethical Considerations 

All data used for this study were obtained during the realization of TETIS study and according with the protocol of this trial (16). TETIS study complied with the principles laid down by the 18th World Medical Assembly of Helsinki in 1964 and all applicable amendments laid down by the World Medical Assemblies and was performed according to the applicable regulations (European directive 2001/20/EC and Spanish Royal Decree 223/2004, of February 6, 2004). The protocol of TETIS study was approved by the Ethical Committee of the Dentistry Hospital-Barcelona University and the Medicine Spanish Agency. All patients provided their Written Informed Consent for the obtention of the data used in this work. This informed consent was also reviewed and approved by the Ethical Committee of the Dentistry Hospital-Barcelona University (University Campus of Bellvitge).

## Results

-Demographic data and characteristics of subjects included in the study

Of the 24 patients 13 were women and 11 were men. The mean age of the participants was 35.2 (rank 18 to 54). Fifteen subjects (62.5%) presented previous concomitant diseases, 54.17% (13 patients) were regular smokers and five patients were regular drinkers (20.8%). An additional total of eight patients were considered occasional drinkers. The main cause of dental extraction was caries (58.3%) followed by radicular remains (33%). None of the patients showed increased risk of bleeding based on the questionnaire carried out, with a score of below 10 for all patients. Seven subjects (29.2%) had some clinically relevant alterations in the oral mucous membrane ( Table 1).

**Table 1:**
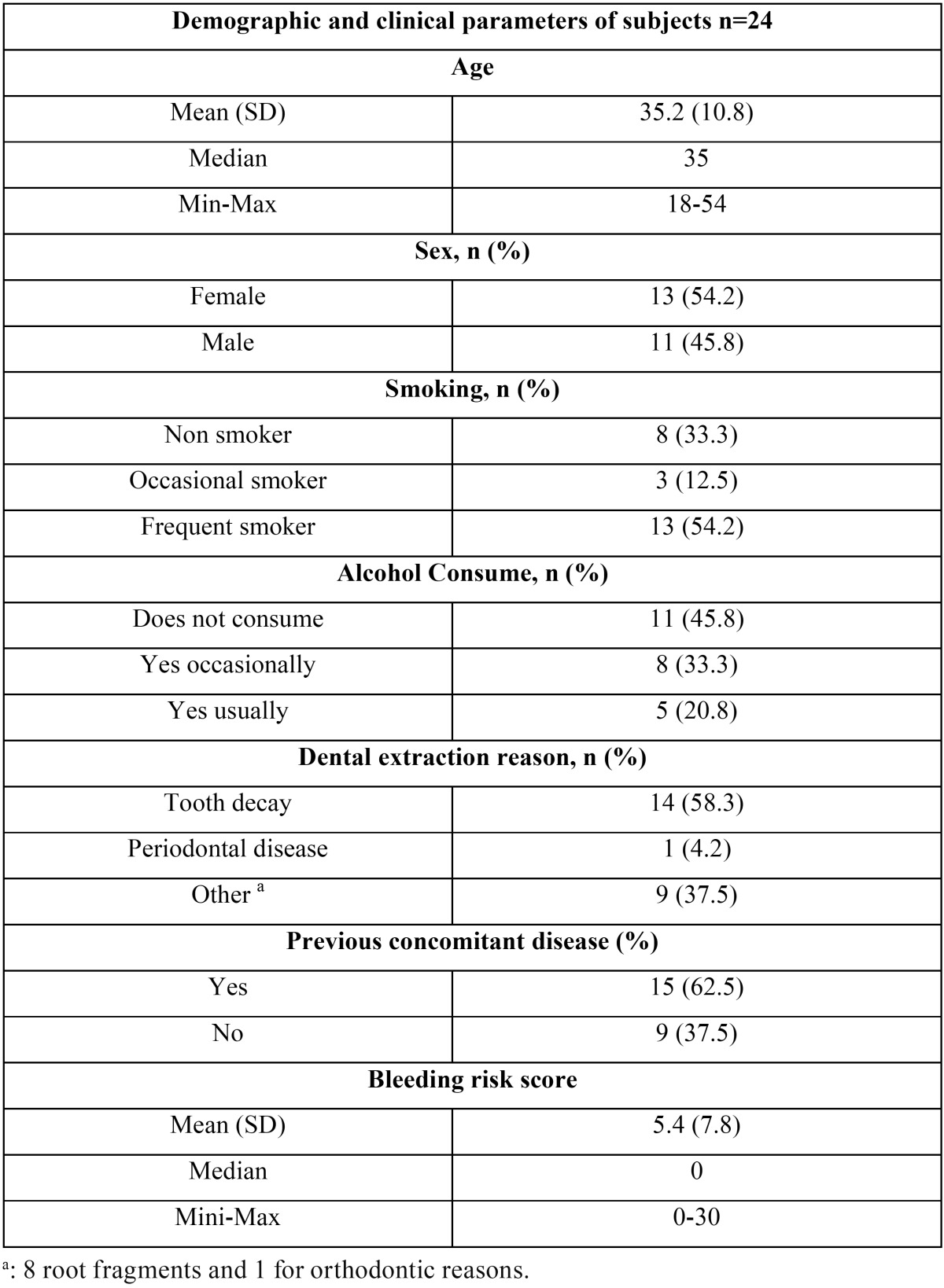
Subject characteristics.

-Vital Signs

**Figure 1:**
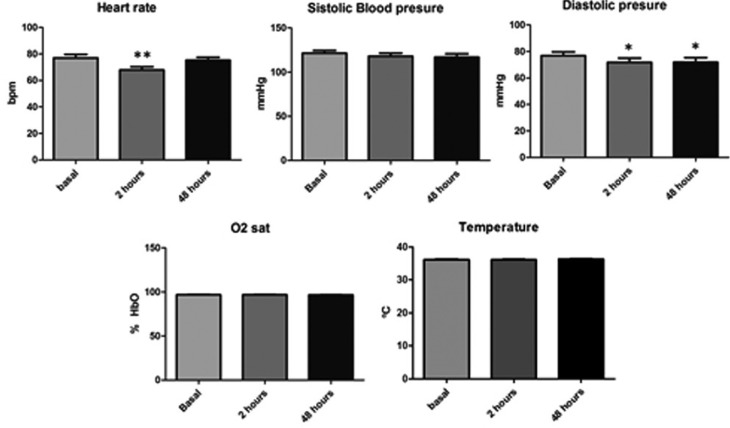
Vital signs of subjects; * *p*<0.05 Bonferroni’s post-hoc test.

The average pre-extraction systolic blood pressure was 121.4±15.1 mmHg (mean±SD) and that of the diastolic blood pressure was 76.9±13.4 mmHg. The body temperature was 36.18±0.5 ºC and the oxygen saturation level was of 97.07±2.13 %. Diastolic pressure was significantly lower (*p*<0.05) at 2 and 48 hours after extraction (71.8±15.9 and 71.8±16.9 respectively). In the same way, the heart rate was significantly lower at 2 hours after extraction (77±13.2 in front of 67.8±12.9 bpm; *p*<0.01) ( Table 2 and Fig. 1).

**Table 2:**
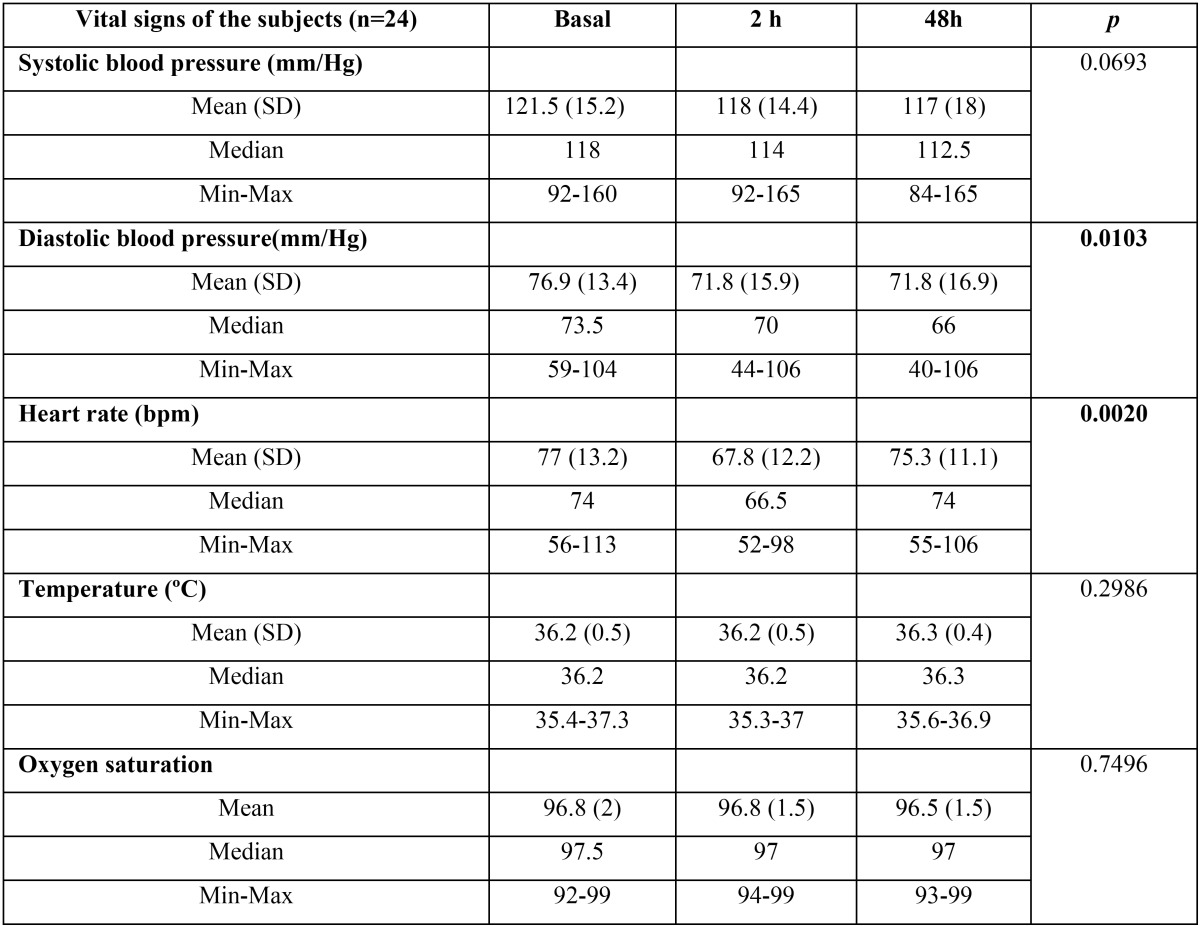
Vital signs of the subjects.

-Blood Test parameters 

**Figure 2:**
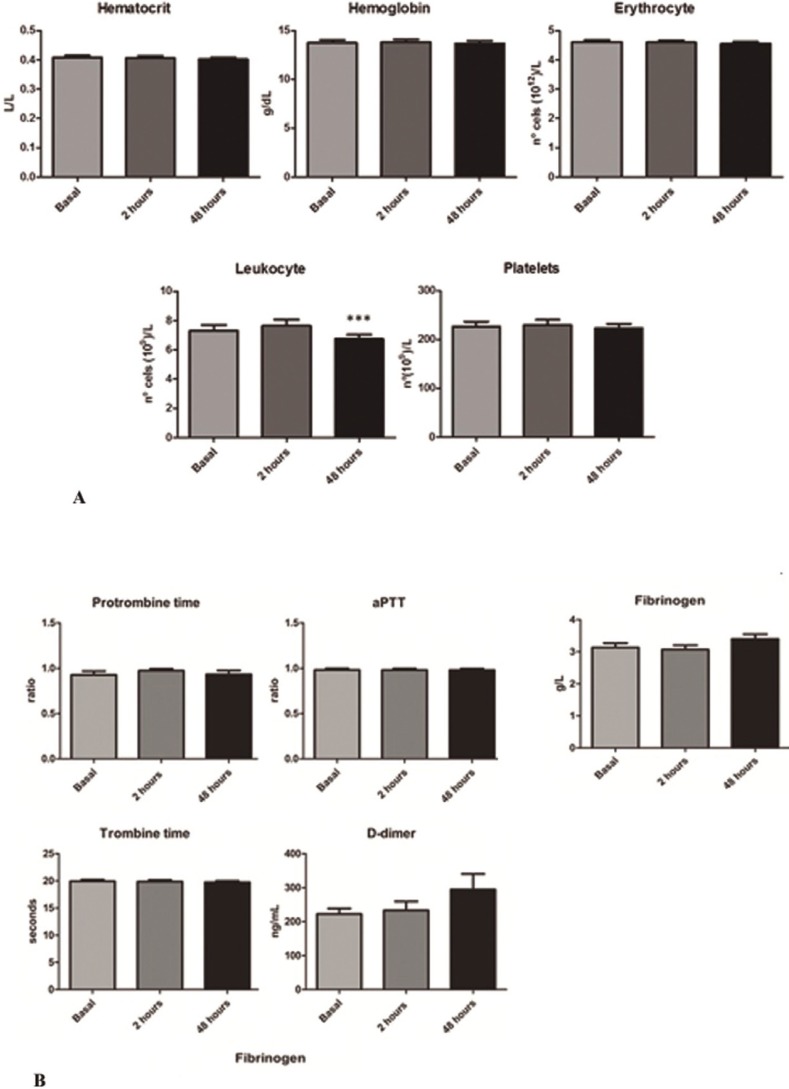
A) Hemogram results obtained. * *p*<0.05 Bonferroni’s post-hoc test. B) Results of biochemistry serum parameters. * *p*<0.05 Bonferroni’s post-hoc test.

**Figure 3:**
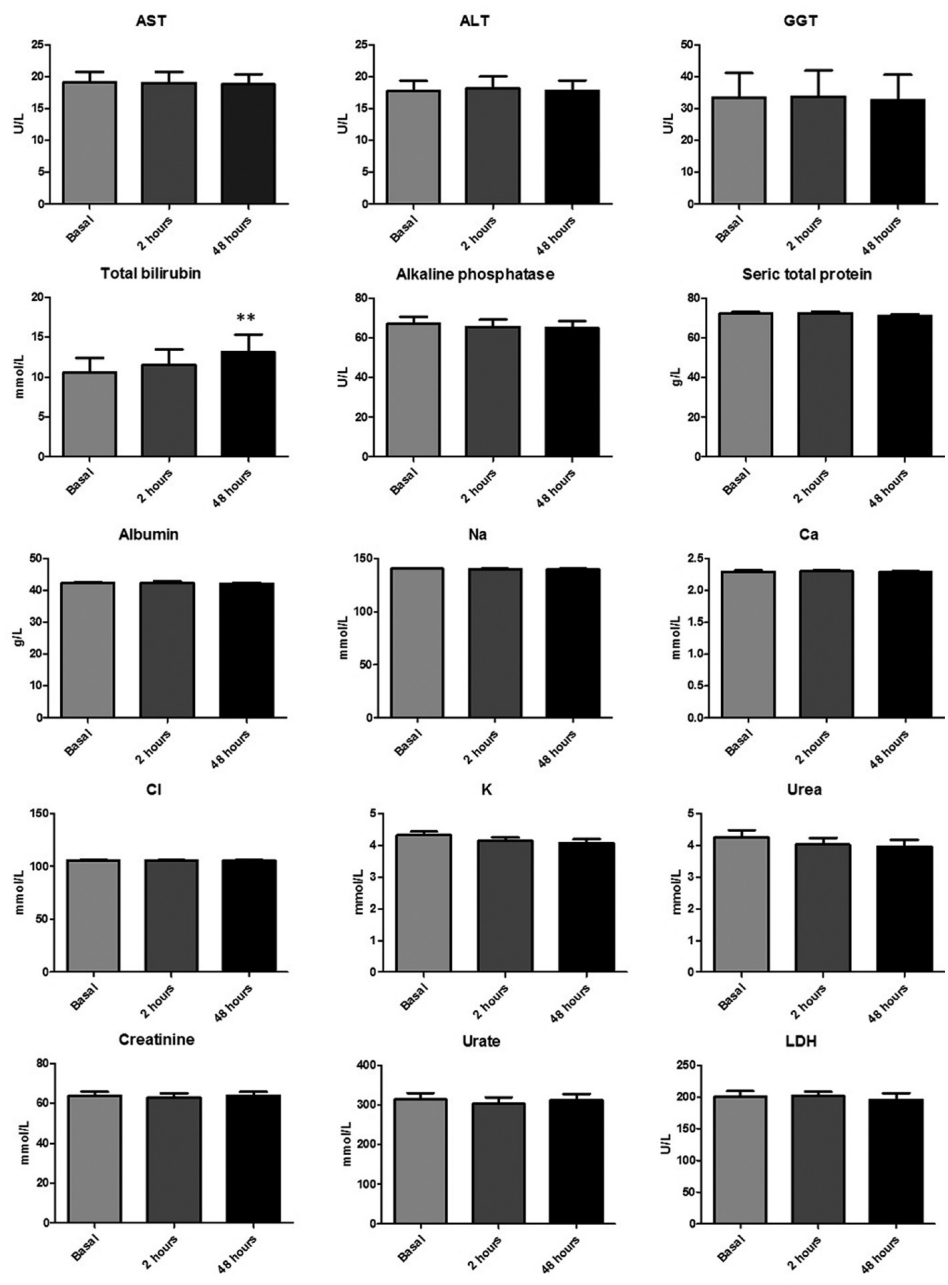
Result of coagulation parameters determined in the study.

The hemogram values and coagulation profile were essentially identical between basal determination and those obtained at 2 and 48 hours after tooth extraction, with the only exception of leukocyte count that was significantly lower at 48 hours (7.3±1.8 in front of 6.7±1.4x109/L; *p*<0.001) ( Table 3 and Fig. 2A). Similar pattern was observed by the biochemistry parameters determined in serum. However, a non-significant increase in D-dimer and fibrinogen was observed at 48 hours after extraction. In fact the mean values obtained at basal determination were 221.7±76.1 ng/mL and 3.1±0.6 g/L respectively. At 48 hours the mean values obtained were 293.6±202.5 ng/mL for the D-dimer and 3.4±0.7 g/L for the fibrinogen ( Table 3 and Fig. 2B). In the same way, an increase in mean total bilirubin levels at 48 hours after extraction was also observed (10.5±9.17 in front of 13.13±10.79 mmol/L). In this case the increment reach the statistical significance (*p*<0.01) ( Table 3 and Fig. 3).

**Table 3:**
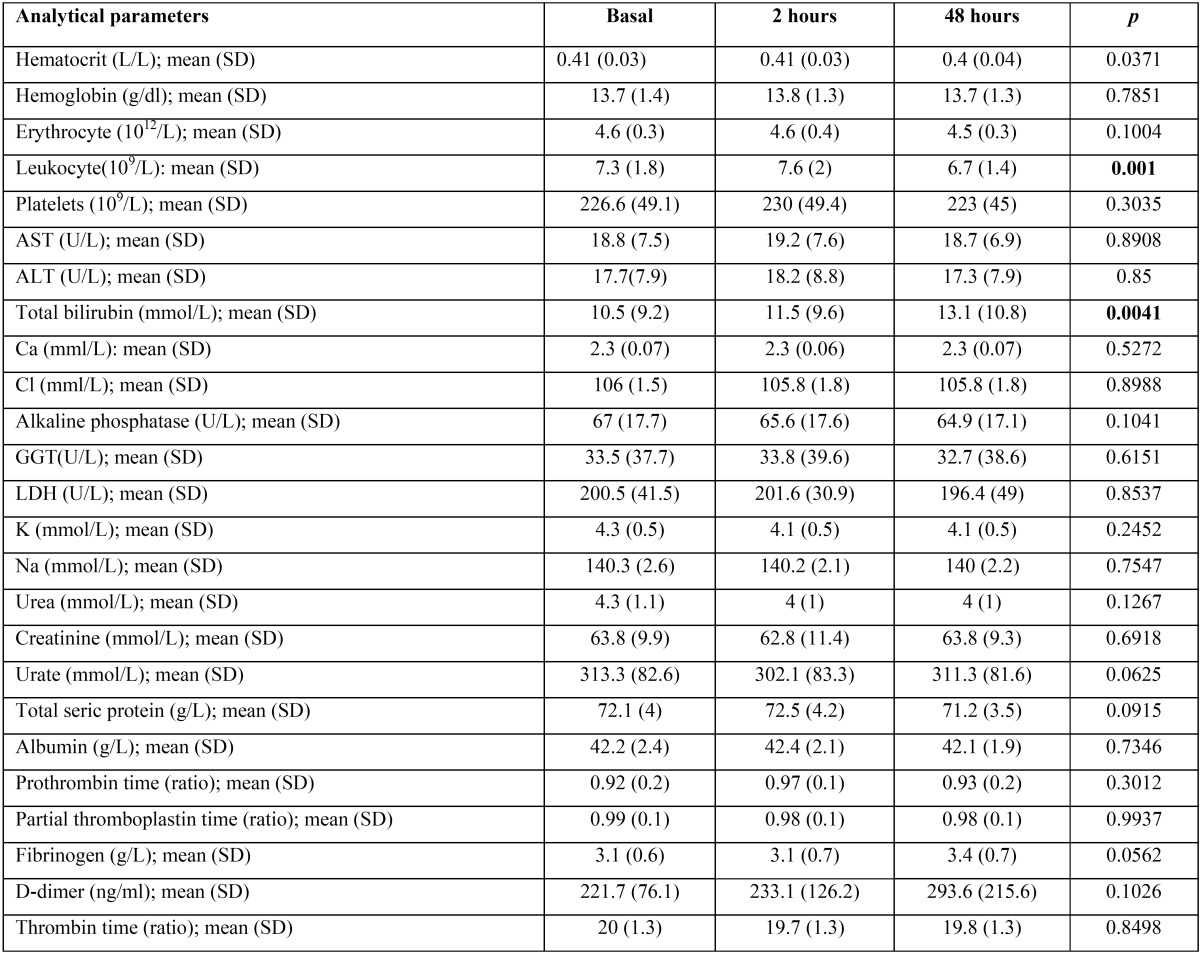
Analytical parameters of the subjects.

## Discussion

The male to female ratio does not represent a bias if we take into account the objective of this research and the intraindividual variation analysis. The average age of 35.2 was justified by the need to have the participation of healthy adults in the phase I study. With regard to tobacco and alcohol consumption in our sample, there is greater proportion of smokers than in general population according to the official report of the Spanish Department of Health’s Survey: (http://www.msssi.gob.es/estadEstudios/estadisticas/encuestaNacional/encuesta2006.htm), or the proportion reported by others (17). Due to the trial’s exclusion criteria, we did not recruit subjects with previous relevant pathology that could have an effect on the parameters of the study. In respect of the reason for extraction, if we keep in mind that periodontal disease tends to appear after the age of 30, the percentage of deep periodontal pockets was only 3.9% in the 35 to 45 age group. In contrast, the prevalence of caries in this age range is much higher. Therefore it is understandable that majority of the extractions were due to caries or complete destruction of the crown (22 teeth out of 24). Only one tooth was extracted due to orthodontist’s recommendation.

In regard to vital signs, we have not found significant variations between the systolic blood pressure, body temperature and SatO2 obtained before and after the dental extraction. In contrast, diastolic blood pressure was significantly lower at 2 and 48 hours after the procedure. In the same way, we observed a significantly lower heart rate at 2 hours ( Table 2 and Fig. 1). However, the magnitude of these changes was very low and always in the normality rank and cannot be considered physiologically relevant. The majority of research papers that evaluated blood pressure in oral surgery do so based on the type of anesthesia used. In this way, the use of Articaine 4% was compared to that of Bupivacaine 0.5% (both with Epinephrine 1:200,000) in 18 patients who underwent a third molar extraction (18). This study reported a higher average systolic pressure at the beginning of the surgery in the Articaine group that returned to normal values after the osteotomy. The oxygen saturation levels showed no differences between the groups or throughout the treatment. Heart rate was also higher in the Articaine group. Another study that tried to control anxiety before the extraction of third molars through music, found that the vital signs significantly changed throughout the surgery in accordance with the stage of the procedure, reaching a maximum peak at the time of the initial incision, and then rapidly decreasing and becoming stabilized within the normal limits (19). In regard to blood pressure, there was no difference between the two groups, but there was such difference for the level of anxiety (*P* <0.05). There were minor changes in the cardiac frequency that were not significant in the music group.

More clear changes were reported by other authors that monitored the cardiovascular signs in a group of 25 healthy patients who underwent a simple extraction. The evaluation took place in four phases: baseline situation, post-anesthesia (Mepivacaine 2% with 1:100,000), post-extraction and upon finishing. The authors observed an increase in systolic and diastolic blood pressure as well in heart rate during the procedure (9).

In contrast with these studies, the average blood pressure observed in our study was lower ( Table 2 and Fig. 1). This could be related to the lower average age of our population. Moreover, as opposed to the significant findings described by other authors we did not observed increments in the heart rate or systolic blood pressure related with the intervention. This could be explained by the time when the measurements were performed. In our case, the first one was performed at 2 hours after the end of the extraction. This time interval could be enough to normalize the increments in cardiovascular parameters induced by the extraction or anesthesia.

Likewise, an interesting paper evaluated the changes in blood pressure related to local anesthesia with various epinephrine concentrations (20). They were able to find a significant increase of systolic blood pressure after injection on the group where lidocaine 2% with epinephrine 1:80,000 was used when compared to the group where articaine 4% with epinephrine 1:200,000 was applied. In a similar way, significantly higher figures throughout the surgery, both with respect to blood pressure as well as cardiac frequency has been reported (14). The average age of patients of this study was higher, 48 years old, and 22% of the participants presented hypertension. In a similar study, moderate figures of hypertension (above 160/110 mmHg) were found in 42.35% of the sample, but the average age in this case was 60.5 years (21). In this study the authors did not find any significant difference between the use, or lack thereof, of vasoconstrictor with respect to the levels of blood pressure, cardiac frequency and oxygen saturation. To our knowledge, no other studies regarding the impact of dental extraction on blood analytical parameters have been reported. We observed that D-dimer, which is directly related to clot reabsorption and tissue injury, showed a very slight non-significant increment at 48 hours after extraction. A similar tendency was observed in the case of fibrinogen. Some authors have found significant variations for this parameter in relation with periodontal treatment, but other authors did not observe variations even in complex maxillofacial surgeries (22,23). Finally, a small significant increment in total bilirubin was observed at 48 hours after the extraction, which could be related with the absorption of hemoglobin contained in the clot. However, taking into consideration the small amount of hemoglobin contained in a clot formed as a result of dental extraction, it is highly probable that this finding was simply a type one statistical error secondary to the small sample size of the study. Similar explanation could be applied to the lower leukocyte count observed at this time point ( Table 3 and Fig. 2A).

## Conclusions

Our results indicate that a simple dental extraction does not induce physiologically relevant changes in vital signs and analytical parameters at 2 and 48 hours after procedure.
